# Learning from the first: a qualitative study of the psychosocial benefits and treatment burdens of long‐acting cabotegravir/rilpivirine among early adopters in three U.S. clinics

**DOI:** 10.1002/jia2.26394

**Published:** 2024-11-20

**Authors:** Katerina A. Christopoulos, Mollie B. Smith, Priyasha Pareek, Alicia Dawdani, Xavier A. Erguera, Kaylin V. Dance, Ryan S. Walker, Janet Grochowski, Francis Mayorga‐Munoz, Matthew D. Hickey, Mallory O. Johnson, John Sauceda, Jose I. Gutierrez, Elizabeth T. Montgomery, Jonathan A. Colasanti, Lauren F. Collins, Moira C. McNulty, Kimberly A. Koester

**Affiliations:** ^1^ Department of Medicine University of California San Francisco San Francisco California USA; ^2^ University of Massachusetts Chan Medical School Worcester Massachusetts USA; ^3^ Department of Medicine Emory University Atlanta Georgia USA; ^4^ Department of Medicine University of Chicago Chicago Illinois USA; ^5^ Ponce de Leon Center Grady Health System Atlanta Georgia USA; ^6^ Columbia University Vagelos College of Physicians and Surgeons New York New York USA; ^7^ Family Health Care Nursing University of California San Francisco San Francisco California USA; ^8^ Research Triangle International Berkeley California USA; ^9^ Department of Epidemiology and Biostatistics University of California San Francisco San Francisco California USA

**Keywords:** adherence, antiretroviral agents, cabotegravir, injections, rilpivirine drug combination, viral suppression

## Abstract

**Introduction:**

Perspectives on long‐acting injectable cabotegravir/rilpivirine (CAB/RPV‐LA) from HIV health disparity populations are under‐represented in current literature yet crucial to optimize delivery.

**Methods:**

Between August 2022 and May 2023, we conducted in‐depth interviews with people with HIV (PWH) at four HIV clinics in Atlanta, Chicago and San Francisco. Eligibility criteria were current CAB/RPV‐LA use with receipt of ≥3 injections or CAB/RPV‐LA discontinuation. We purposefully sampled for PWH who initiated with viraemia (plasma HIV RNA >50 copies/ml) due to adherence challenges, discontinuers, and cis and trans women. Interviews were coded and analysed using thematic methods grounded in descriptive phenomenology. Clinical data were abstracted from the medical record.

**Results:**

The sample (San Francisco *n* = 25, Atlanta *n* = 20, Chicago *n* = 14 for total *n* = 59, median number of injections = 6) consisted of 48 PWH using CAB/RPV‐LA and 11 who had discontinued. The median age was 50 (range 25–73) and 40 (68%) identified as racial/ethnic minorities, 19 (32%) cis or trans women, 16 (29%) were experiencing homelessness/unstable housing, 12 (20%) had recently used methamphetamine or opioids and 11 (19%) initiated with viraemia. All participants except one (who discontinued) had evidence of viral suppression at interview. Typical benefits of CAB/RPV‐LA included increased convenience, privacy and freedom from being reminded of HIV and reduced anxiety about forgetting pills. However, PWH who became virally suppressed through CAB/RPV‐LA use also experienced an amelioration of feelings of shame and negative self‐worth related to oral adherence challenges. Regardless of baseline viral suppression status, successful use of CAB/RPV‐LA amplified positive provider/clinic relationships, and CAB/RPV‐LA was often viewed as less “work” than oral antiretroviral therapy, which created space to attend to other aspects of health and wellness. For some participants, CAB/RPV‐LA remained “work,” particularly with regard to injection site pain and visit frequency. At times, these burdens outweighed the aforementioned benefits, resulting in discontinuation.

**Conclusions:**

CAB/RPV‐LA offers a range of logistical, psychosocial and care engagement benefits, which are experienced maximally by PWH initiating with viraemia due to adherence challenges; however, benefits do not always outweigh treatment burdens and can result in discontinuation. Our findings on rationales for persistence versus discontinuation can inform both initial and follow‐up patient counselling.

## 1 INTRODUCTION

Long‐acting injectable cabotegravir and rilpivirine (CAB/RPV‐LA) every 4 or 8 weeks is the first complete long‐acting antiretroviral therapy (ART) regimen for the treatment of HIV. While approved by the United States Food and Drug Administration (FDA) only for people with HIV (PWH) with stable viral suppression, data from small clinic cohorts supports use in PWH with viraemia due to adherence challenges [1, 2, 3]. In February 2024, a data safety monitoring board stopped a randomized trial of CAB/RPV‐LA in PWH with adherence challenges because it demonstrated superior efficacy to oral ART [4]. In March 2024, IAS‐USA guidelines endorsed use in this population when supported by intensive follow‐up and case management [5]. Indeed, CAB/RPV‐LA can mitigate challenges to ART adherence that range from individual‐level factors (e.g. psychological burden of daily pill‐taking), to comorbid conditions (e.g. mental illness, substance use), to interpersonal, community and societal forces (e.g. HIV‐related stigma).

In registrational trials and industry‐sponsored implementation projects, PWH receiving CAB/RPV‐LA have endorsed psychosocial benefits, namely increased psychological and logistical freedom, while acknowledging concerns about injection pain and efficacy [6, 7, 8, 9]. However, these studies had low numbers of female, Black/African American and Latine PWH, and did not account for adverse socio‐structural determinants of health, such as housing instability, substance use and mental illness. Perspectives from these groups, who carry the greatest burden of HIV in the United States, are necessary to optimize the effective and equitable delivery of these novel agents in the real world [10]. Further, little is known about the experience of PWH who discontinue CAB/RPV‐LA, which can contribute important lessons learned to implementation efforts.

In addition, studies to date have not included perspectives of PWH unable to achieve or maintain viral suppression on oral ART but who become suppressed through the use of CAB/RPV‐LA. Understanding how and why PWH with adherence challenges experience clinical success on CAB/RPV‐LA can better guide treatment decision‐making in this population.

The objective of this study was to fill existing gaps in the literature by exploring the lived experience of early adopters of CAB/RPV‐LA, focusing on psychosocial impact and motivations for persistence, in a racially, ethnically and gender‐diverse sample of PWH who face social and structural challenges to treatment engagement.

## 2 METHODS

### 2.1 Study design and research team

This cross‐sectional qualitative study utilized one‐time semi‐structured one‐on‐one in‐depth interviews. The core research team consisted of a physician lead at all sites (KAC, MCM, JAC, LFC), a medical anthropologist (KAK), research coordinators with qualitative experience (XAE, MBS, RSW, KVD, AD) and a third‐year medical student (PP). Interviewers were not previously known to participants.

### 2.2 Study setting and participants

The study took place in four urban academic HIV clinics: Ward 86 in San Francisco, the Grady Ponce de Leon Center and the Emory Midtown Infectious Disease clinic in Atlanta, and the University of Chicago. Ward 86 and Ponce are safety‐net providers for PWH with public insurance or who are uninsured. The Atlanta and Chicago clinics serve the majority of Black/African‐American patients and comparatively more cisgender women, while Ward 86 cares for a higher proportion of gay‐identified men and PWH experiencing homelessness and substance use. During the study (2022−2023), the Chicago clinic was offering CAB/RPV‐LA under FDA‐label guidelines, the Atlanta clinics were not fully scaled and offering CAB/RPV‐LA to PWH with viraemia only in select cases and the San Francisco clinic was conducting a demonstration project of off‐label use of CAB/RPV‐LAB in PWH with viraemia due to adherence challenges.

### 2.3 Eligibility criteria, sampling and recruitment

Study eligibility criteria were age ≥ 18 years, English or Spanish‐speaking and having received ≥3 CAB/RPV‐LA injections (to ensure sufficient time to reflect on potential impacts) or having discontinued CAB/RPV‐LA at any point. We purposefully sampled for those initiating CAB/RPV‐LA with viraemia using the threshold from registrational trials [11, 12, 13] (HIV plasma RNA >50 copies/ml), which reflects off‐label use of CAB/RPV‐LA, individuals who discontinued CAB/RPV‐LA and at least 30% women, given that few U.S. women were represented in qualitative studies from clinical trials [8]. We made one exception to enrol a participant who had had only two injections as he was a high‐priority informant per sampling criteria (initiating with viraemia). Patients were offered participation by clinic staff either during reminder calls for injection appointments or at the injection visit, or by their primary HIV provider during a clinic visit or via telephone or secure messaging.

### 2.4 Data collection

Informed by a socio‐ecological model of care engagement [14], the interview guide asked about experience living with HIV and oral ART, motivations for CAB/RPV‐LA uptake, the process of starting, receipt of injections, reflections on CAB/RPV‐LA use as compared to oral ART and attitudes towards other long‐acting ART formulations. When applicable, questioning explored the shift from every 4 to every 8‐week dosing and reasons for discontinuation. The guide was piloted with three patients for content, flow, and yield and revised iteratively.

Between August 2022 and May 2023, coordinators conducted 59 interviews either in person in a private space near the clinic or via video‐conference. Interviews were audio‐recorded and lasted 60–90 minutes, after which participants completed a socio‐demographic survey and received $50 compensation for their time. Coordinators wrote field notes to capture impressions about the interview and performed medical record review to abstract plasma HIV RNA and CD4 cell count measurements, time on injections, current dosing schedule (every 4 or every 8 weeks) and number of injections. Participants were considered virally suppressed at the time of the interview if plasma HIV RNA measurement was <200 copies/ml, as this threshold typically determines an effective response to ART [15]. Interviews were professionally transcribed and uploaded into Dedoose (Version 9.0.107, Socio‐Cultural Research Consultants, Los Angeles, CA) to facilitate data management and retrieval.

### 2.5 Data analysis

The analytic approach followed three stages typical of thematic analysis grounded in descriptive phenomenology [16]. In the first stage, *familiarization*, the research team created an analysis table with headings derived from guide questions as well as topics of importance identified from transcripts, for example psychosocial impact, privacy considerations. Coordinators tabled data and wrote brief analytic summaries of each case, identifying interviews with rich descriptions. The core researchers discussed interviews as they occurred with the larger investigative team on weekly study calls and reviewed the analytic table to see whether cases were adding new information or aligning with previously identified topics of importance, aiding the determination of saturation.

In the second stage, *exploration*, the team discussed a subset of individuals with viral suppression versus viraemia at CAB/RPV‐LA initiation, concentrating on narratives of self‐management of living with HIV and taking daily ART and the psychosocial impact of CAB/RPV‐LA. This discussion resulted in a focused codebook with seven broad codes. Two coordinators coded each transcript, resolving differences through discussion. Three analysts (KAC, MBS, RSW) read and discussed the excerpts labelled “consideration and uptake” and “psychosocial impact” to identify emerging themes.

In the third stage, *recontextualization*, the core research team sought to add nuance and depth to findings by re‐reading transcripts to recontextualize emerging themes within individual narratives. Using a structured template, analyst pairs considered the participant holistically, described how psychosocial impacts unfolded and critically assessed how the case aligned or diverged from emerging themes. Using the analytic table, the team chose 19 cases to template and discuss, beginning with cases who exemplified findings published in literature from clinical trials, including cases who voiced minimal impact on their lives, followed by cases who described significant impacts on mental health and wellbeing, and ending with cases of discontinuation. This intensive cross‐case comparison resulted in an analytic memo (KAK, KAC) that further honed the insights reported in this paper.

The Consolidated Criteria for Reporting Qualitative Research was used to report study methods and findings [17]. The interview guide, codebook and structured template are available in Files S2–S4. Participant quotes are annotated with “VS” to indicate those who initiated CAB/RPV‐LA virally suppressed and “NVS” to indicate those initiating with viraemia due to adherence challenges.

### 2.6 Ethical approval

The University of California San Francisco Institutional Review Board performed an ethical review of this study under the single‐site institutional review board requirement of the National Institutes of Health. Participants provided written informed consent.

## 3 RESULTS

### 3.1 Participant characteristics

We interviewed 48 PWH currently using CAB/RPV‐LA and 11 who had discontinued (Table 1 and Table S1). The median age was 50 (range 25–73), 14 (24%) were cisgender women, 5 (9%) were transgender women, 28 (48%) were Black/African‐American and 6 (10%) were Latine. With regard to self‐reported psychological and socio‐structural vulnerabilities, 16 (29%) had housing instability, 12 (20%) had used methamphetamine or opioids in the past 30 days, 27 (46%) reported psychiatric illness (e.g. depression, anxiety, bipolar disorder, schizophrenia) and 24 (41%) endorsed financial difficulties. Eleven participants (19%) initiated CAB/RPV‐LA with viraemia. The median number of injections was 6 (IQR 4,9) with a median time on injections of 256 days (IQR 195,347), with 51% on q8week dosing. All participants initiating CAB/RPV‐LA with viraemia were on q4week dosing, as were all those who discontinued except for one. All participants were virally suppressed at the time of the interview except for one participant who initiated CAB/RPV with viral suppression and subsequently discontinued; at interview, this participant reported being off ART and had no plasma HIV RNA measurement after discontinuation.

**Table 1:** Characteristics of participants receiving long‐acting injectable cabotegravir/rilpivirine (CAB/RPV) in routine care

	Total sample *N* = 59	Currently on CAB/RPV‐LA *N* = 48	Discontinuers *N* = 11
Site			
Ward 86	25 (42%)	20 (42%)	5 (45%)
Ponce De Leon	10 (17%)	8 (17%)	2 (18%)
Midtown	10 (17%)	7 (15%)	3 (27%)
University of Chicago	14 (24%)	13 (27%)	1 (9%)
Age, median (minimum/maximum)	50 (25−73)	50 (26−73)	50 (25−68)
Age			
18–29 years	6 (10%)	5 (10%)	1 (9%)
30–49 years	23 (39%)	19 (40%)	4 (36%)
≥50 years	30 (51%)	24 (50%)	6 (55%)
Time period of diagnosis			
After 2021	3 (5%)	3 (6%)	0 (0%)
2007–2020	26 (44%)	20 (42%)	6 (55%)
1997–2006	13 (22%)	10 (21%)	3 (27%)
Before 1997	17 (29%)	15 (31%)	2 (18%)
Gender identity			
Male	38 (64%)	30 (63%)	8 (73%)
Female	14 (24%)	13 (27%)	1 (9%)
Transgender female	5 (9%)	4 (8%)	1 (9%)
Other^a^	2 (3%)	1 (2%)	1 (9%)
Race			
White	19 (32%)	15 (31%)	4 (40%)
Black	28 (47%)	25 (52%)	3 (30%)
Multiracial/other	11 (19%)	8 (17%)	3 (30%)
Hispanic ethnicity	6 (10%)	4 (8%)	2 (18%)
Heterosexual	22 (37%)	20 (43%)	2 (18%)
Housing			
Own/rent	42 (71%)	34 (71%)	8 (73%)
Single room occupancy/hotel	11 (19%)	10 (21%)	1 (9%)
Staying with friends/family	6 (10%)	4 (8%)	2 (18%)
Reported methamphetamine or intravenous drug use in the past 30 days	12 (21%)	8 (17%)	4 (36%)
Self‐reported mental health diagnosis	27 (46%)	24 (50%)	3 (27%)
Education			
At least some college/post‐graduate studies	39 (67%)	32 (68%)	7 (67%)
High school graduate/General education development test	11 (19%)	10 (21%)	1 (9%)
Less than high school	8 (14%)	5 (11%)	3 (27%)
Financial situation			
Comfortable, have money for extras	10 (17%)	9 (20%)	1 (9%)
Have money for necessities	23 (39%)	16 (35%)	7 (64%)
Barely paying the bills	14 (24%)	12 (26%)	2 (18%)
Struggling to survive	10 (17%)	9 (20%)	1 (9%)
History of incarceration	22 (38%)	19 (40%)	3 (27%)
Reported the use of other daily oral medications	40 (68%)	32 (67%)	8 (73%)
Plasma HIV‐RNA at CAB/RPV‐LA initiation			
≥50 copies/ml (viraemic)	11 (19%)	8 (17%)	3 (27%)
<50 copies/ml (virally suppressed)^b^	48 (81%)	40 (83%)	8 (73%)
Log^10^ plasma HIV‐RNA in those viraemic at CAB/RPV‐LA initiation, mean (SD)	4.36 (1.08)	4.55 (0.83)	3.84 (1.70)
CD4 cells/mm^3^ at CAB/RPV‐LA initiation, median (IQR)			
Viraemic at initiation	442 (99, 616)	475 (20, 996)	244 (99, 616)
Virally suppressed at initiation	694 (472, 890)	679 (66, 2887)	733 (460, 958)
CAB/RPV‐LA dosing interval			
Every 4 weeks	28 (47%)	18 (38%)	10 (91%)
Every 8 weeks	31 (53%)	30 (62%)	1 (9%)
Number of injections at interview, median (IQR)	6 (4, 9)	7 (4, 10)	3 (1, 6)
Self‐reported late injections	6 (10%)	4 (9%)	2 (20%)
Plasma HIV‐RNA <200 copies/ml at interview^c^	58 (98%)	48 (100%)	10 (91%)

*Note*: Data missing for those currently on CAB/RPV‐LA: Hispanic ethnicity (*n* = 2), sexual orientation (*n* = 1), drug use (*n* = 1), education (*n* = 1), financial situation (*n* = 2), incarceration history (*n* = 1), late injections (*n* = 1). Data missing for discontinuers: race (*n* = 1), late injections (*n* = 2), viral load at interview (*n* = 1).

All participants initiating CAB/RPV‐LA with plasma HIV‐RNA >50 copies/ml were on q4week dosing.

All participants who discontinued CAB/RPV‐LA were on q4week dosing except one.

Abbreviations: CAB/RPV‐LA, long‐acting cabotegravir/rilpivirine; HIV‐RNA, human immunodeficiency virus ribonucleic acid; IQR, interquartile range; ml, millilitre; SD, standard deviation.

^a^ Other self‐reported gender identities: “gender‐queer” and “male‐ish.”

^b^ Includes participants with plasma HIV‐RNA levels of “Not Detected,” “Detected <30 copies/ml” (UCSF) and “Detected <20 copies/ml” (Ponce de Leon, Midtown and University of Chicago).

^c^ One participant did not have a follow‐up viral load at the time of interview and reported being off oral ART.

### 3.2 Qualitative results

The major finding was that the psychosocial impacts of CAB/RPV‐LA stemmed from a participant's prior relationship to their HIV status and experience with daily oral ART adherence. We observed a range of benefits that depended on the extent of antecedent logistical, psychological, social and care engagement burden conferred by oral ART. For those who did not express internalized HIV stigma or ART adherence challenges, CAB/RPV‐LA had minimal psychosocial impact, with the primary benefits manifesting as increased convenience and reduced anxiety about forgetting daily pills. Those with concerns about inadvertent HIV status disclosure from oral ART welcomed the privacy afforded by CAB/RPV‐LA. For those expressing internalized HIV stigma and/or burden from living with a chronic disease, removing pills as a daily reminder of HIV status was described as liberating and allowed them to feel more “normal.” Individuals who struggled to adhere to oral ART, and particularly those who initiated CAB/RPV‐LA with viraemia, described marked psychosocial benefits, specifically reduced shame and improved self‐worth, as these participants reported they were finally able to achieve “good patient” status, which the literature defines as being compliant with, grateful for and coping well with HIV treatment [18, 19, 20].

**Figure 1:**
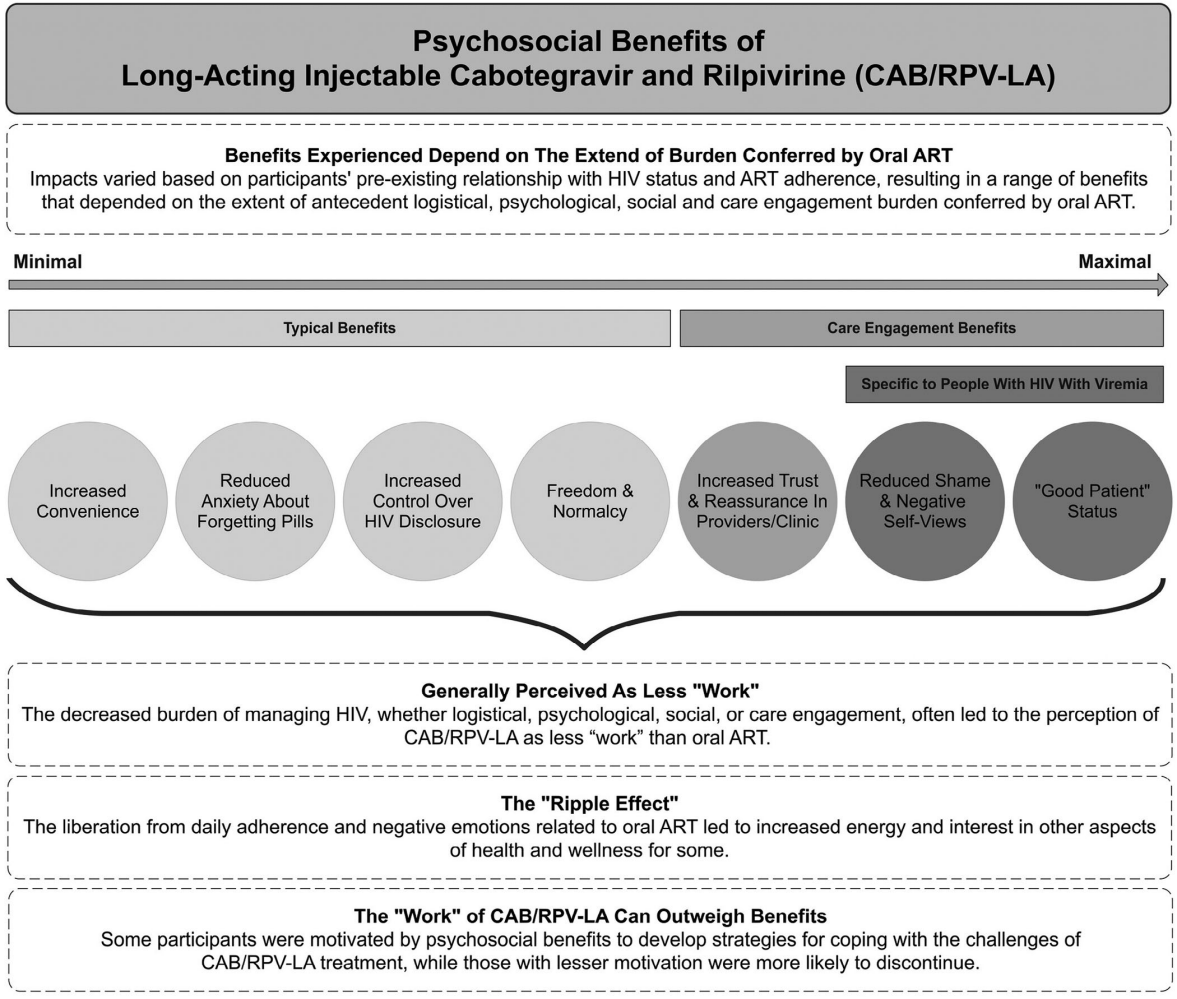
Psychosocial benefits of long‐acting cabotegravir and rilpivirine (CAB/RPV‐LA).

In addition to the finding that the antecedent burden of oral ART determined the range of benefits experienced with CAB/RPV‐LA, we identified three themes (Figure 1). First, successful use of CAB/RPV‐LA amplified positive aspects of relationships with providers and the clinic, regardless of viral suppression status at baseline. Second, the reduction in the logistical, psychological, social or care engagement burden of managing one's HIV frequently resulted in a view of CAB/RPV‐LA as less “work” than oral ART, which created space to attend to other aspects of health and wellness. However, it is important to note that CAB/RPV‐LA remained “work” for some participants, particularly with regard to injection site pain and frequency of visits. A third theme was that benefits did not always outweigh the burden, resulting in discontinuation.

### 3.3 Typical psychosocial benefits include logistical and psychological freedom

A typical case reported increased convenience and reduced worry about daily pill‐taking.

*As I mentioned, you know, not having to worry about taking a pill. Or even remembering, did I take the pill? You know, you just get the injection and keep it moving. And you know, it's like you have so much time on your hands and you don't have to worry about lugging around pills. Or even having to run to the pharmacy to get a prescription refilled…It's like it gives you time back*.

*40YO mixed‐race bisexual man (VS), Atlanta*

These participants tended to prefer the every 8‐week dosing option, especially because of increased freedom to travel. As the participant quoted above noted, “It was like every time I look up, I've got to get back [to the clinic]…I definitely appreciate the bimonthly a lot more….” Some of these participants noted minimal impact on their lives beyond increased convenience.

The sense of psychological freedom with CAB/RPV‐LA use was conveyed via phrases like “*load lifted from my shoulders*,” “*weight off my back*” and “*peace of mind*.” As one participant stated:

*And also the psychological aspect of it. It's like being imprisoned in a space that you can't leave. You're locked in this room and you're pulling on the door, but you can't open it. That feeling of being imprisoned. There is no way escaping this daily routine of so many pills per day. There is no freedom from it*.

*66YO White gay man (VS), Chicago*

Importantly, the reduced anxiety around daily oral pill‐taking was accompanied by increased confidence in having consistent levels of ART in one's body.

*There's a sense of security with the injection that I didn't have with the pill. Because I never knew whether I was going to test undetectable or not because of throwing up so much… I didn't know if enough was staying inside of me. I never had that security*.

*61YO White heterosexual woman (VS), San Francisco*

However, one participant did acknowledge that shifting to CAB/RPV‐LA led to a feeling of loss of control with regard to taking care of his HIV, whereas taking daily oral ART reassured him the medication was present in his system and working.

### 3.4 Increased privacy and sense of normalcy

The benefit of increased privacy around HIV status was common, and also associated with the perception that it was less work to prevent inadvertent HIV disclosure than with oral ART. As a 28‐year‐old VS Black gay man in Atlanta stated, “I like the fact that it gives more room to be discreet…I used to scrape my name off of the pill bottle because you never know where your trash is going….” Interestingly, some participants mentioned that injection marks and soreness left by CAB/RPV‐LA functioned as visible markers of adherence to intimate partners, which a 30‐year‐old Latine man (VS) in San Francisco stated was “more believable and creates more trust” in the context of undetectable = untransmittable (U = U) [21].

The relief from not having to take a daily pill was also framed as freedom from being reminded of one's HIV status, as if one were living without HIV, which resulted in feeling more “normal.” The young Latine man who described pill taking as, “a little bit of trauma every day” said “it [CAB/RPV‐LA] does make a big difference.” As a 38‐year‐old Black trans woman (VS) from Atlanta stated, “Like it's much easier for me to feel as if I don't have it and I'm just a normal person, that I just take a maintenance drug, such as insulin.”

### 3.5 Maximal psychosocial benefit is experienced by PWH who become virally suppressed

We observed a powerful easing of negative self‐views resulting from difficulties adhering to oral ART. While some participants who initiated CAB/RPV‐LA with viral suppression expressed feeling better about their adherence behaviour with injections, those who experienced this benefit to the greatest extent were PWH who became virally suppressed with CAB/RPV‐LA. One participant experiencing homelessness and using stimulants described the impact it had on him as follows:

*Really, mostly, it was the shame and guilt. That was it. It was like, “Ah! I don't have to have the shame face. You don't have to be guilty. You don't have to take this pill or be remembering to take the pill.” And “When my bloodwork comes back, you know, it'll be good because I did what I'm supposed to do.” Mostly, it's me doing what I'm supposed to do on my part for my health*.

*43YO Black gay man (NVS), San Francisco*

Indeed, some of these participants noted a shift away from maladaptive behaviours. For example, one participant who struggled with adherence stated that he was always hearing bad news about his health at visits with his provider. After initiating CAB/RPV‐LA, he described no longer having to use substances to cope with coming to the clinic.

*I would know that I wasn't going to be able to take the medication, so I would get high before…. Now that everything's better, everything's working, it's not so difficult to come here. It's so much easier because I know my health is right…. I'm happy to come in, and it's a better feeling. And I don't be high before I come here*.

*32YO Black gay man (NVS), San Francisco*

This sense of redemption was also experienced through overcoming self‐stigma, especially with regard to perceptions of oneself as unclean and able to transmit virus to others. A 49‐year‐old White heterosexual man in San Francisco (NVS) described how achieving undetectable status was meaningful for him because he previously felt “contagious,” as though he were “dangerous” and “dirty.”

### 3.6 Increase in positive attitudes towards providers and the clinic

While PWH initiating CAB/RPV‐LA with viraemia often noted that their adherence to injections and resultant viral suppression allowed them to achieve good patient status, some of those starting CAB/RPV‐LA with stable viral suppression saw it as a reward for having been a good patient, that is by having adhered to oral ART. As expressed by a 61‐year‐old Black heterosexual woman (VS) in Atlanta. “It's like, ‘Wow!’ It's a pot of gold at the end of the rainbow.”

In both situations, simultaneously attending to one's health and meeting provider expectations created a positive feedback loop, increasing shared good feeling and resulting in favourable attitudes towards clinic attendance. This point was articulated clearly by a 30‐year‐old Black gay man (VS) in Chicago, who stated, “It's kind of become something I'm looking forward to, you know? I'm coming to take care of my health and at the same time, I'm coming to get some great positive energy from some positive individuals.” As a 51‐year‐old White gay man (NVS) in San Francisco noted, “I would be happy, the doctor would be happy… Everybody is going to be happy.”

### 3.7 Decreased “work” of HIV self‐management creates space to attend to health and wellness

The reduced cognitive load from not having to procure and take daily oral pills was expressed as less “work” involved in managing one's HIV.

*That whole, constantly having to, keep all that stuff in my head is – I'm so glad that I don't have to do that. In fact, it's a lot less work because it's only one trip down here. Instead of all of the mental math, and the driving down to get pills every month.’’*

*40YO White gay man (VS), Atlanta*

The sense of liberation from the work and negative emotions related to daily ART adherence resulted in a “ripple effect,” in which some participants described increased energy and interest in other aspects of their health and wellness. As a result of CAB/RPV‐LA, a 55‐year‐old Black heterosexual woman (NVS) in San Francisco reported being more social, volunteering at a local food pantry and working towards her general educational development test (GED). “I've been getting out more. Doing a lot more things.”

This “ripple effect” of psychosocial benefits was most pronounced for participants who became virally suppressed by using CAB/RPV‐LA. As one participant stated:

*I see a psychiatrist once a week, my therapist once a week. I take much less pills. I take my bipolar medication, sleeping medication, but no more HIV meds. My stomach is much, much better. I stopped drinking. I broke up with this relationship. I'm forming different habits. You know, I go to AA (Alcoholics Anonymous) Meetings, I go support groups. It did take lots of anxiety away. Lots of anxiety… I'm not worried about dying. You know, it's more hopeful about my future. There's less stress. I stress people out less. So I think the impact is huge*.

*51YO White gay man (NVS), San Francisco*

While the metaphor of “work” was commonly used to describe the effort required to take oral ART, it is worth noting that some participants did not see oral ART or CAB/RPV‐LA through this lens.

*It [CAB/RPV‐LA] comes first…it's a priority. [Like] traveling, for gas, it's just something that I have to deal with. I fit it into my schedule like I fit everything else in there*.

*40YO Black heterosexual woman (VS), Chicago*

These participants described ART as a commitment, and once they committed, they would persist, even if they found aspects of the experience challenging. This experience contrasted with what we observed among individuals who elected to discontinue the use of CAB/RPV‐LA because it was too much work.

### 3.8 The work of CAB/RPV‐LA can outweigh benefits, resulting in discontinuation

**Figure 2:**
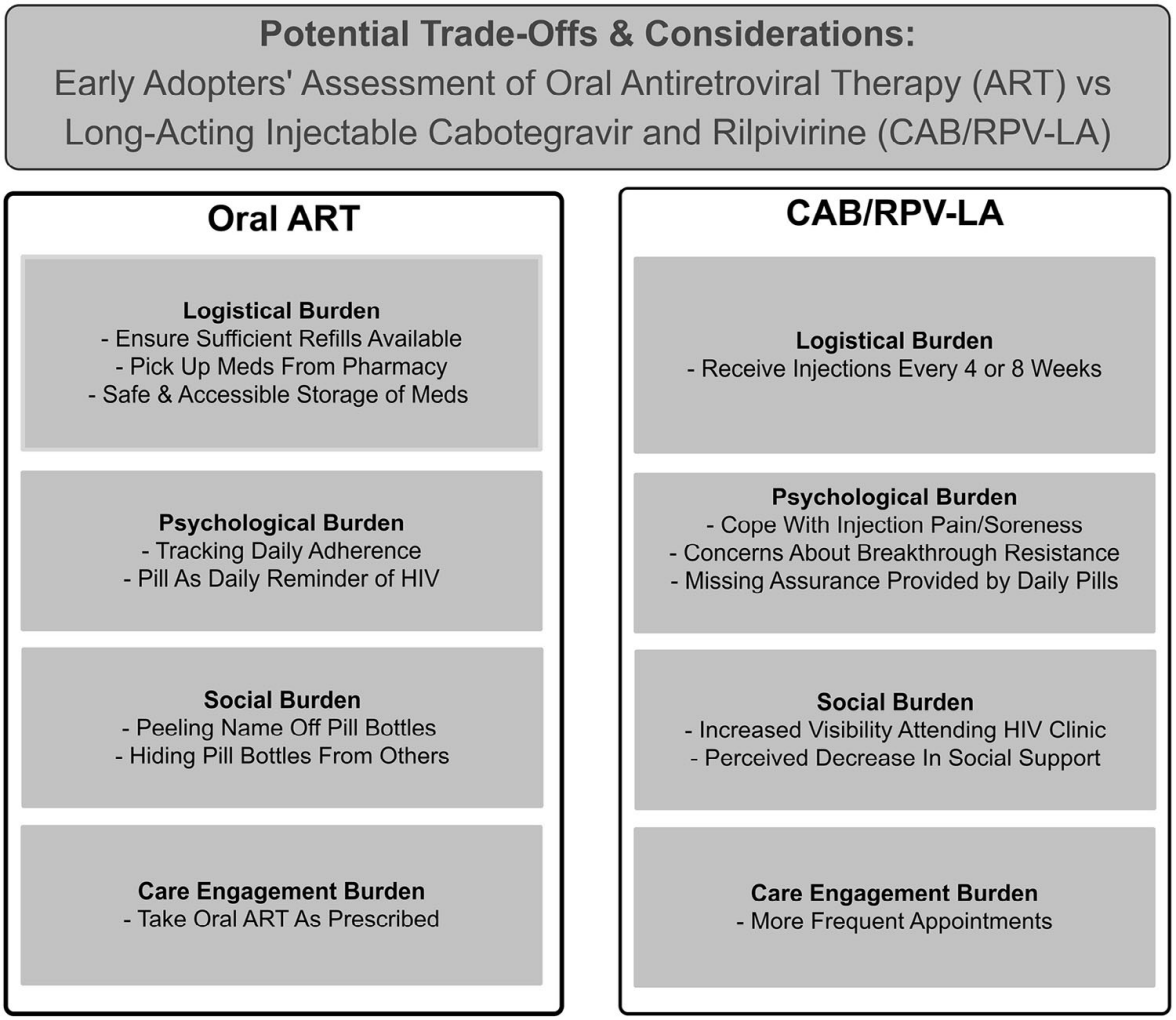
Potential trade‐offs and considerations: early adopters’ assessment of oral ART versus CAB/RPV‐LA.

Notably, some participants viewed CAB/RPV‐LA as work. For example, the participant who had experienced oral ART as a “little bit of trauma every day” found it effortful to come to the clinic, but for him, the trade‐off was worth it, as he felt more “normal.” In our dataset, the biggest amount of work associated with CAB/RPV‐LA was “gearing up for” and experiencing pain, both during injections and in the following days. Participants for whom the benefits of CAB/RPV‐LA tipped the scales developed strategies to manage this pain, for example walking after injections, while those with less incentive discontinued. For example, a 48‐year‐old VS Latine gay man in San Francisco without adherence challenges or privacy concerns who still had to take other daily medications found that several weeks of “freedom” did not outweigh managing injection pain, stating “It was just not worth it to be suffering 50% of the time.” Figure 2 summarizes trade‐offs in CAB/RPV‐LA use.

There were two participants who initiated CAB/RPV‐LA with viraemia who discontinued it due to injection pain (and in one case, abscesses). Interestingly, for these participants, both of whom had transiently experienced viral suppression with oral ART in the past year, the experience of CAB/RPV‐LA gave them a greater appreciation for and drive to persist with oral ART. As one 43‐year‐old White gay man (NVS) in San Francisco stated, “I felt that pain. I was like, ‘Oh gosh. I can't wait to get back on that pill. I'm going to take those pills and stay on it.’” A 40‐year‐old multi‐racial gay non‐binary individual (NVS) in San Francisco reported that they would rather “deal with the pill, because it agrees with my body more and it gives me something to have a habit about.” Indeed, this participant did not view coming to the clinic every 4 weeks as onerous, as they liked the routine and enjoyed seeing their provider and other staff. When asked by the interviewer, each participant acknowledged that they were aware that CAB/RPV‐LA could stay in the body for up to a year after discontinuing and that taking oral ART was important to prevent the emergence of resistant virus due to declining drug levels.

Of note, in several cases, the decision to discontinue occurred in isolation from HIV providers, as participants simply resumed their oral ART as advised, but without notifying the clinic. These participants noted that the first several CAB/RPV‐LA injections occurred without an associated provider visit. While participants greatly appreciated the support provided by pharmacists and nurses, some expressed a desire for more contact with one's HIV provider early in the course of injections. As one participant stated:

*I felt lost. When I'm on the shot, it's like basically no one cares anymore because I wasn't being seen by a doctor anymore, wasn't getting my blood drawn or anything. It just – things didn't feel right…*.

*25YO Latine gay man (VS), Chicago*

This participant received injections in the hospital infusion unit because of requirements dictated by insurance determination of CAB/RPV‐LA as a medical, rather than a pharmacy benefit. He described not going to the clinic for his injection visit as a loss of an important source of support.

*They [the clinic] would basically speak to you a little bit, see how you're doing, how have you been, what new things have you been doing, stuff like that. And then you finally speak to your doctor. With Cabenuva, it was completely different. You had to go one long hallway, then to another long hallway, meet with a security guy, then say you're for infusions, and they tell you go down this hallway, then you're going to take the elevator to go up….You're basically on a little map search to get your shots. And then once you get there, they just check you in right away, what's your name, and then you just wait, get your shot, and go*.

This participant's decision to discontinue CAB/RPV‐LA after about 5 months was multifactorial; in addition to not seeing his doctor and perceiving that his CD4 cell count and HIV viral load were not being monitored, he reported injection site pain, a “golf‐ball size mass,” weight gain and recurrence of chronic depression. While he did resume oral ART, he stated during the interview that he was not currently taking it and expressed ambivalence about having stopped CAB/RPV‐LA.

Indeed, at times the decision to discontinue CAB/RPV‐LA was accompanied by a negative psychological impact. A 50‐year‐old White gay man (VS) in Atlanta who “very regretfully” decided to stop due to injection site pain stated that he felt like a “loser.” Several other participants reported feeling guilty when they were aware of the effort the clinic had made to obtain the medications.

All participants who discontinued expressed potential interest in other long‐acting formulations when queried. For some, this interest hinged on the availability of another modality, such as a long‐acting pill or patch, while others noted that they would be willing to tolerate the pain of injections if the injections were less frequent, at least several months apart.

## 4 DISCUSSION

In this first qualitative study of the lived experience of CAB/RPV‐LA use in routine care among a diverse sample of PWH in the United States, including those initiating CAB/RPV‐LA with viraemia due to adherence challenges, we found a range of psychosocial benefits. Similar to findings from industry trials [6, 7, 8, 9], these benefits included increased logistical and psychological freedom from not having to take a daily pill, easing of concerns around inadvertent disclosure of HIV status and no longer being reminded of one's HIV status daily.

However, our analysis is unique in that it situates the use of CAB/RPV‐LA in a participant's HIV and ART adherence history, considers the role of adverse socio‐structural determinants of health in narratives of treatment adherence and includes PWH who initiated CAB/RPV‐LA with viraemia. As such, our study highlights that the extent of the psychosocial benefits experienced with CAB/RPV‐LA depended on the extent of barriers to daily oral pill‐taking. Specifically, PWH whose barriers previously prevented them from achieving viral suppression on oral ART experienced maximal impact via improved self‐worth, which was highly motivating to persist with using CAB/RPV‐LA, in contrast to hypothetical provider concerns about the use of CAB/RPV‐LA in PWH with adherence challenges [22].

We also noted that regardless of viral suppression status at baseline, meeting physician expectations for successful use of CAB/RPV‐LA created positive impacts on HIV care engagement [23]. The benefits of using CAB/RPV‐LA were commonly viewed through a lens of this novel therapy being less “work” than oral ART and as a result, some participants reported increased energy and motivation to attend to other aspects of their health and wellness. These positive impacts help explain the treatment success observed in studies of CAB/RPV‐LA in PWH with viraemia from adherence challenges [3, 24].

Our analysis also adds to the literature in that, unlike interviews done with trial participants, we report on the experience of those who discontinued CAB/RPV‐LA. Here, we found a trade‐off between the psychological, logistical and physical impacts of CAB/RPV‐LA. Those for whom the benefits continued to outweigh the burden developed strategies to cope with the less desirable aspects of injections, while others found the burden too great and discontinued. Importantly, those who discontinued remained open to trying other formulations of long‐acting ART, including pills and patches. Similar to advances in contraception and the evolving context of methods for HIV pre‐exposure prophylaxis [25, 26], it stands to reason that having multiple options to meet an individual's preferences and life circumstances can improve the likelihood of treatment success.

Indeed, the cases of discontinuation illustrate that CAB/RPV‐LA is not always experienced as easier than or preferable to oral ART. While most participants said they received counselling about potential side effects, a few wished they had been better prepared for pain at and after injections. These cases highlight the importance of anticipatory guidance for pain and also reinforce that patient‐provider discussions of CAB/RPV‐LA should carefully explore motivations for uptake, and support patients in identifying the objectives they wish to achieve with the use of CAB/RPV‐LA. The spectrum of benefits identified in this study offers providers a way to frame some of these goals vis a vis logistical, psychological and privacy concerns. Particularly for individuals without adherence challenges or experiences of HIV stigma, careful weighing of anticipated benefits against potential burdens can optimize shared decision‐making, especially for those at all apprehensive about injections. While ideal for the primary HIV provider to engage patients in these discussions, these efforts could be assisted by others involved in CAB/RPV‐LA delivery, for example clinic pharmacists and nurses, potentially with formal decision aids [27]. In addition, coupling this counselling with other health initiatives based on life circumstances, for example long‐acting treatments for alcohol use disorder, opioid use disorder or contraception, could offer an effective way to approach an individual's health goals more holistically [28, 29].

Importantly, some participants wished for more contact with their primary HIV provider early on in receiving injections. Joint discussions throughout the early initiation period to identify emerging benefits and potentially address burdens of CAB/RPV‐LA could help support efforts to persist versus safely discontinue, and individuals with histories of depression or anxiety may require extra support. Finally, as some patients may experience negative self‐worth upon discontinuation, providers should be prepared to provide reassurance that CAB/RPV‐LA may not be the right fit for everyone and that it is acceptable to switch back to oral ART if desired or clinically necessary. Building in the ability for rapid consultation with one's primary HIV provider about the experience of CAB/RPV‐LA use may have merit as an important aspect of clinic programmes.

The strengths of our study were a diverse multi‐site sample, including from the U.S. South, a region with a disproportionate burden of HIV, and intentionally focusing on those who initiated CAB/RPV‐LA with viraemia as well as those who discontinued. These perspectives enabled a richer exploration of the lived experience of CAB/RPV‐LA than previously presented. Limitations include that nearly all participants who started with viraemia were from San Francisco due to site‐specific practices during the study. In addition, our research occurred early in CAB/RPV‐LA implementation; continued inquiry into benefits and burdens will be important, particularly as the number of people who discontinue may increase and we did not reach saturation on this experience due to the small number who had discontinued at the time of data collection. Participants were receiving care in urban clinics; perspectives of CAB/RPV‐LA users in rural areas should be explored. While we had the capacity for interviews in Spanish, few monolingual Spanish speakers were receiving CAB/RPV‐LA during the study; further dedicated study of CAB/RPV‐LA use in this group may be warranted. Finally, we do not present analyses by socio‐demographic and clinical characteristics of interest.

## 5 CONCLUSIONS

In summary, in a sample of PWH from San Francisco, Atlanta and Chicago, we found that CAB/RPV‐LA offers a range of logistical, psychosocial and clinical benefits, which are experienced maximally by those initiating with viraemia; however, benefits do not always outweigh treatment burdens. Understanding the varied reasons for persistence and discontinuation can help optimize the delivery of this important treatment option.

## COMPETING INTERESTS

Dr. Christopoulos and Dr McNulty have been advisory board members for Gilead Sciences outside the proposed work. Dr. Christopoulos has been a workshop participant for Janssen.

## AUTHORS’ CONTRIBUTIONS

KAC, KAK and XAE designed the study. JG, FM‐M and MDH were essential in the recruitment of patients initiating CAB/RPV‐LA with viraemia due to adherence challenges. MBS, PP, AD, XAE, KVD and RSW collected the data, and MBS, AD, XAE, KVD and RSW coded interview transcripts. KAC, MBS, PP, AD, XAE, KVD, RSW, JAC, LFC, MCM and KAK analysed the data, with MDH, ETM, MOJ, JS and JIG providing ongoing critical intellectual feedback. KAC wrote the paper. All authors have read and approved the final manuscript.

## FUNDING

This study was supported by R01 MH123396 (KAC). LFC is supported by K23 AG084415.

## Supporting information




**File S1**: Table S1

**File S2**: Early Adopter Interview Guide

**File S3**: Early Adopter Codebook

**File S4**: Early Adopter Analysis Template

## Data Availability

Data are not publicly available due to privacy concerns. Data from code excerpts are available on request from the corresponding author.
