# Supplementary material for: Learning from the first: a qualitative study of the psychosocial benefits and treatment burdens of long‐acting cabotegravir/rilpivirine among early adopters in three U.S. clinics
Source: J Int AIDS Soc. 2024 Nov 20;27(11):e26394. doi: 10.1002/jia2.26394 (PMC11578930; doi:10.1002/jia2.26394)
Supplement: Supplementary file 1 — File S1: Table S1 [file JIA2-27-e26394-s001.docx]

Supplementary Table 1. Plasma HIV-RNA Measurements for Participants with Viremia at Initiation

|  | Plasma HIV-RNA at CAB/RPV-LA Initiation (copies/mL) | Plasma HIV-RNA at Interview (copies/mL) |
| --- | --- | --- |
| 1 | 118,000 | <30 |
| 2 | 74,900 | ND |
| 3 | 173,900 | <30 |
| 4 | 11,600 | <30 |
| 5 | 10,600 | ND |
| 6 | 8,500 | <30 |
| 7 | 2,300 | <30 |
| 8 | 693,900 | <30 |
| 9 | 123,800 | <30 |
| 10 | 32,900 | ND |
| 11 | 80 | ND |

Measurements >100 rounded to nearest 100

HIV-RNA = human immunodeficiency virus ribonucleic acid; CAB/RPV-LA = long-acting cabotegavir/rilpivirine; mL= milliliter; ND = Not detected
