# Supplementary material for: Learning from the first: a qualitative study of the psychosocial benefits and treatment burdens of long‐acting cabotegravir/rilpivirine among early adopters in three U.S. clinics
Source: J Int AIDS Soc. 2024 Nov 20;27(11):e26394. doi: 10.1002/jia2.26394 (PMC11578930; doi:10.1002/jia2.26394)
Supplement: Supplementary file 2 — File S2: Early Adopter Interview Guide [file JIA2-27-e26394-s004.docx]

**Opening and Warm-Up**

Let’s start with some simple questions.

1. Could you please start by telling me a little about yourself?
   1. How do you feel about discussing Cabenuva today?

**Background Information:**

I would like to learn a little bit more about you and your situation.

1. Where are you from originally?
   1. What brought you to [Name of City]?
2. Tell me about your living situation. Where and with whom do you live?
   1. How long has that been the case?
3. How did you get here today?
   1. Is this how you usually get to and from your appointments? If not, how do you usually get to and from the clinic on days you have appointments?

**HIV Journey Experience:**

I’d like to change topics a little bit and ask a few questions abouts your relationship with HIV.

1. How long have you been living with HIV?
2. Tell me about your experience getting medical care for HIV.
3. Tell me about your experience with medication for HIV **before long-acting injectables.**
   1. In general, how has being on HIV treatment gone for you? Any side-effects?
   2. Tell me about your experience with trying to become undetectable (UD)? What does being undetectable mean to you?
4. Who in your life knows about your HIV status?

**Consideration of Long-Acting**

Now I would like to focus on the main topic we hope to discuss – your use of long-acting injectable HIV medications; the rest of the interview will focus on this topic.

1. Think back to the first time you heard about LAI. Tell me about that moment.
   1. What was your initial reaction? What was going through your mind?
   2. How did you feel, learning that something like LAI-ART might be available to you?
2. Think back to the first time you talked to someone else about LAI. Tell me about this moment.
   1. Why this person? Who brought it up? What information was shared?
3. How else did you go about getting more information about LAI? Be as specific as possible.
   1. Did you do any independent research? If yes, what did you learn or take away from it? How was the information similar or different than what you had already heard?
   2. If applicable; How did you learn that you could get the shot every 2 months? Why did you think this was right for you? Did you have any concerns?
4. What made you want to switch to (or start, if not previously on treatment) LAI? Walk me through your thinking and logic.
5. What appealed to you about LAI?
6. What did not appeal to you about LAI?
   1. If they mention what did not appeal, ask: What made it worth it to you to start?

**Uptake of Long-Acting**

Now we want to know the actual process you went through to get on it. We want to hear what actually happened so we can understand better what is working and what could be improved.

1. Tell me about the discussion you had with your doctor where it was decided that you would you be moving forward with LAI. Potential Probes: What was that conversation like? Had you discussed LAI previously? Were there any hold ups? What helped? Was it a shared decision?
   1. Did you talk to any other clinic staff and other providers about LAI to get you ready to start? If yes, who else did you talk to and what did you talk about?
   2. Did you talk to the pharmacy staff? If yes, how was that? What role did the pharmacy play in helping to get you ready?
2. Tell me about what you took away from the instructions around coming in for injection visits. Potential Probe: What did you learn about the timing of injections?
   1. If window/flexibility is mentioned ask: How does this timing/window/level of flexibility make you feel? How close to the limit have you gotten?
3. Tell me about what you took away from the conversation around potential side-effects or problems with LAI? Potential Probes: How big of a concern is or was this for you?
   1. If side-effects were concerning, ask: What helped ease this concern?
4. What, if anything, did you learn from the clinic about developing resistance with late or missed injections? Potential Probe: How big of a concern is or was this for you?
   1. If developing resistance was concerning, ask: What helped ease this concern?
5. How long did the process take from the point of talking with your provider and when you finally received the first injection? Was this about what you expected? Tell me about that.
   1. What, if anything, helped move the process forward?
   2. What kinds of barriers, if any, did you encounter?
6. What kinds of concerns did you or do you still have around the cost of this medication?
   1. Who helped you with insurance issues like making sure you had the right coverage?
   2. Have any unusual insurance issues come up since starting? Tell me about those.
   3. Do you have a co-pay? If yes, how much do you pay for each injection and visit?
7. Optional: Did you transition from your prior antiretrovirals to the oral Cabenuva/Rilpiverine medications before receiving the first injection?
   1. How was that decision made?
   2. Possible probes: How long did you take oral Cabenuva/Rilpiverine? Did you miss any doses?

**Experience of Long-Acting**

Now we want to focus on your experience in more detail. We will start with the first visit and then talk more generally about how it has been going and any issues that have come up.

1. How did the first appointment for getting your injections go?
2. What was going through your mind at that time? How were you feeling?
3. Was there anything you did not expect or that surprised you?
4. What, if anything, could have made your first injection visit better?

**[Skip To Q23 for Q4-to-Q8 Switches – Alternative to Q22, General Experience on Shot]**

**[Skip To Q24 for Discontinuation Cases – Alternative to Q22, General Experience on Shot]**

1. How have the injections been going in general?
2. Have you had any side effects? If yes, tell me how does it/do they impact your life?
3. How long does an injection visit typically last? Including travel, what are your thoughts on the current amount of time you are dedicating to getting your injection on-time?
4. [Ask Switch Cases] How have the injections been going in general?
   1. Have you had any side-effects? If yes, tell me how does it/do they impact your life? Did the side-effect factor into your decision to switch? How big of a factor was it? If you had fewer side-effects, would you have stayed on the monthly version? Why or why not?
   2. How long does an injection visit typically last? Did the time spent getting LAI influence your decision to switch to every two-months? How big of a factor was it? If visits were shorter, would you have stayed on the monthly version? Why or why not?
5. [Ask Discontinuation Cases] What was it like being on injections for HIV?
   1. Did you have any side-effects? If yes, tell me how did it/did they impact your life? Did the side-effect factor into your decision to stop? How big of a factor was it? If you had fewer side-effects, would you have stayed on the injection? Why or why not?
   2. How long did an injection visit typically last? Did the time spent getting LAI influence your decision to stop? How big of a factor was it? If visits were shorter, would you have stayed on the injection? Why or why not?

**[Skip To Q26 for Q4-To-Q8 Switches – Alternative to Q25, Visit Frequency & Quality]**

**[Skip To Q27 for Discontinuation Cases – Alternative to Q25, Visit Frequency & Quality]**

1. How often do you come in for injections? How many injections have you received to date?
   1. What is it like to come to the clinic every month? If applicable, what is it like to come to the clinic every two months?
   2. What thoughts or feelings come to mind when you think about coming in for your shots?
   3. How is this “new normal” similar and different than coming to the clinic previously before you started LAI? How happy are you with this new arrangement?
2. [Ask Switch Cases] How often were you coming in for injections when you first started? How many injections did you get before you switched to every 2-month injections? How many 2-month injections have you gotten to date?
   1. What was it like to come to the clinic every month and then switch to every two months? What changed and what stayed the same going from one to two months?
   2. What thoughts or feelings come up when you think about coming in for your shots? Has this changed since switching to every two months?
   3. How is this “new normal” similar and different than coming to the clinic previously before you started LAI? How happy are you with this new arrangement?
3. [Ask Discontinuation Cases] How often were you coming in for injections when you were still on the shot? How many injections did you get before you stopped?
   1. What was it like to come to the clinic every month? If applicable, what was it like to come to the clinic every two months?
   2. What thoughts or feelings came up when you thought of having to come in for your shots? Has this changed since stopping LAI? Has this changed since stopping the injections?
   3. How was your “time on the shot” similar and different than coming to the clinic previously before you started LAI? How is your care now (“after LAI”) like compared to the past?

**[Skip To Q29 for Q4-To-Q8 Switches – Alternative to Q28, People Involved]**

**[Skip To Q30 for Discontinuation Cases – Alternative to Q28, People Involved]**

1. Tell me about the people that help you get your injections. What’s it like to get care from these individuals, start with the front desk staff and continue in order from there.
   1. Do you see your doctor at any of your injection appointments? What was that like? How has your relationship with your doctor changed, if at all?
2. [Ask Switch Cases] Tell me about the people that help you get your injections. What’s it like to get care from these individuals. Let’s start with the front desk staff and continue in order of who you see in a typical visit.
   1. How often have you seen your doctor since starting injections? What is that like? How has your relationship with your doctor changed, if at all?
   2. Did any of them play a role – positive or negative – in your decision to switch to every 2-month injections? Tell me about this.
3. [Ask Discontinuation Cases] Tell me about the people that helped you get your injections. What was it like to get care from these individuals, start with the front desk staff and continue in order.
   1. How often did you see your doctor while you were on the shot? What was that like? How has your relationship with your doctor changed, if at all?
   2. Did any of them play a role – positive or negative – in your decision to stop? Tell me about this.

**[Skip To Q31 for Q4-To-Q8 Switches – Exclusive to Case, Decision to Switch]**

**[Skip To Q32 for Discontinuation Cases – Exclusive to Case, Decision to Stop]**

1. [Ask Switch Cases] I understand you switched from monthly injections to every 2-month injections. Tell me how that came about.
   1. Who did you talk with when you were still considering switching? How did they help inform your decision?
   2. Was it a logical or emotional decision, or both? Tell me about that.
   3. Right before you switched, what, if anything, did you like about monthly LAI-ART? What did you not like about it? How did switching address these downsides?
   4. Can you imagine a time where you would want to switch back to monthly injections?
2. [Ask Discontinuation Cases] I understand you are no longer getting injections. Tell me how that came about.
   1. Who did you talk with when you wanted to/had to stop taking Cabenuva? How did they help inform your decision?
   2. Was it a logical or emotional decision, or both? Tell me about that.
   3. At the end, what did you like about LAI-ART? What did you not like about it? At what point in the process did you start to feel like it was not worth it?
   4. Could you imagine a time where you would want to go back to being on the shot?

**[Skip To Q33 for Q4-To-Q8 Switches – Exclusive to Case, Process for Switching]**

**[Skip To Q34 - Q36 for Discontinuation Cases – Exclusive to Case, Process for Stopping]**

1. [Ask Switch Cases] What was switching like for you? If I was thinking of doing the same, how should I prepare?
   1. What was it like talking to your doctor about switching? What was going through your mind at that time? How were you feeling?
   2. What was your understanding about the process (what would happen and what you would need to do) for switching? Was there anything you did not expect or that surprised you? How involved or how much effort did it require?
   3. What, if anything, could have made your switching experience better?
2. [Ask Discontinuation Cases] What was stopping LAI like for you? If I was thinking of doing the same, how should I prepare?
   1. What was it like talking to your doctor about stopping the injections? What was going through your mind at that time? How were you feeling?
   2. What was your understanding about the process (what would happen and what you would need to do) for stopping? Was there anything you did not expect or that surprised you? How involved or how much effort did it require?
   3. What, if anything, could have made your stopping experience better?

1. [Ask Discontinuation Cases] What was it like switching back to oral ART after being on the injection?
   1. How soon after stopping LAI did you start the pills? What are you taking now (same or different regimen than before)? How is that going?
2. [Ask Discontinuation Cases] Did your CD4 or VL change because of being on the shot? If yes, was this a factor in stopping?
   1. How soon did you realize your CD4/VL was changing? What was it like to get this news?
   2. How has it been, getting back to your “usual levels” since switching back to oral ART?

**[Skip To Q39 & Q40 for Discontinuation Cases – Alternatives to Q37 & Q38, Persistence Issues]**

1. Tell me about any challenges you have with getting your injection every month (or two months).
   1. What makes it difficult to come in regularly? What makes it easy to come in regularly?
   2. Have you ever thought about going back on oral ART? Tell me about that.
2. What are you supposed to do if you miss an injection appointment?
   1. Have you ever missed or been late to an appointment? What happened on those occasions? Who followed up with you?
   2. Do you have back-up pills at home? If yes, how many do you have? When would you have to use them? Have you ever needed to go back on oral ART?
3. [Ask Discontinuation Cases] Tell me about any challenges you had with getting your injection every month (or two months).
   1. What made it difficult to come in regularly? What made it easy to come in regularly?
4. [Ask Discontinuation Cases] What were you supposed to do if you missed an injection appointment?
   1. Did you ever miss or were late to an appointment? What happened on those occasions? Who followed up with you?
   2. Did you have back-up pills at home? If yes, how many did you have? When were you supposed to use them? Did you ever need to go back on oral ART?

**Psycho-Social Impacts of LAI-ART**

Now we want to focus on how going on LAI-ART has impacted your life overall, not just in terms of managing your HIV.

1. For some people, switching from taking HIV medication every day to coming for injections has been no big deal – it’s just more convenient. While for others, this switch has been a very big deal not just a greater convenience, but it has impacted them emotionally. Please take a minute to consider what it has been like for you.
   1. What were your dreams or hopes for taking LAI? Have they come true?
   2. How did you imagine it would impact your health (physical and mental)?
   3. How did you imagine it would impact your life (socially, sexually, etc.)?

**[Skip To Q43 for Discontinuation Cases – Alternative Q42, Pill Burden and Other Benefits]**

1. How is it for you to have fewer (or no) daily pills? Has it impacted other parts of your life?
2. Has LAI affected your sense of privacy around your HIV? How does it compare to the time before LAI?
3. Has LAI affected your sense of how safe HIV meds are for your body? How does it compare to the time before LAI?
4. Has LAI affected your sense of how much work you do to take care of your HIV? How does it compare to the time before LAI?
5. [Ask Discontinuation Cases] What was it like to have to take fewer or no daily pills? How did it impact your life? How does it feel having to go or choosing to go back on oral ART?
6. Did LAI affect your sense of privacy around your HIV? How did it compare to the time before LAI? Have those feelings changed since going back on oral ART?
7. Did LAI affect your sense of how safe HIV meds are for your body? How did it compare to the time before LAI? Have those feelings changed since going back on oral ART?
8. Did LAI affect your sense of how much work you did to take care of your HIV? How did it compare to before LAI? Have those feelings changed since going back on oral ART?
9. Optional: What kind of conversations have you had with people in your personal life about long acting since starting?

**For individuals who were stopped for reasons beyond their control**:

1. Would you have wanted to stay on LAI if you could? Tell me about that. What emotions came up for you, if any (sadness, anger, happiness, relief, etc.)?

***For individuals who have never been undetectable before going on LAI:***

1. How is it for you to be undetectable? How has it impacted your life? How has it impacted your feelings about your HIV diagnosis?

**Suggestions for Improvement**

Now we want to hear your thoughts on ways to make the experience of being on LAI-ART better.

1. Looking back, what do you wish you had known before starting LAI-ART?
2. How long do you want to stay on LAI? Have you ever thought about going back on oral ART?
3. Do you have any suggestions for how LAI services at your clinic could be improved? Tell me about it.
   1. If multiple rec’s, ask: Which one is the most important? Why or explain your thinking?
   2. [Ask Discontinuation Cases] Would this/these improvements have made it easier for you to stay on LAI? Why or why not?
4. Lastly, would you recommend long acting injectables to a friend? Why/why not?

**Length Between Doses/Alternate Formulation**

Now we want to ask you about different treatment options that are still being tested in clinical trials and different alternatives to traditional clinic-based LAI-ART.

1. First, I’m going to ask you to imagine you could choose an ideal length of time between injections. What would it be?
2. Now imagine you could choose a different way to get long-acting medication. What would it be?
   1. What are your thoughts on a **monthly oral pill**, like a monthly pill, instead of an injection?
   2. What are your thoughts on a **monthly patch** that goes on your skin, instead of an injection?
   3. What are your thoughts on a **yearly implant** under the skin, instead of an injection?
3. Imagine that you could receive long-acting at any place, in any way that you wish. What would be your ideal scenario?
   1. What are your thoughts on self-injection (you overseeing giving yourself the shot)?
   2. What are your thoughts on partner-injection (you overseeing giving yourself the shot with the help of a friend)?
   3. What are your thoughts on home-injection (the clinic comes to you and gives you the shot)?

**Closing**

Before we end, we want to give you an opportunity to bring up any issues that we haven’t discussed yet.

1. We have talked about many aspects of LAI-ART. Is there anything else you would like to share about LAI-ART?
2. Are there any last thoughts you would like to share? Is there any topic that you thought we would discuss that I didn’t ask you about?
3. Do you have any questions for me? What are your thoughts about this interview?
