# Supplementary material for: Learning from the first: a qualitative study of the psychosocial benefits and treatment burdens of long‐acting cabotegravir/rilpivirine among early adopters in three U.S. clinics
Source: J Int AIDS Soc. 2024 Nov 20;27(11):e26394. doi: 10.1002/jia2.26394 (PMC11578930; doi:10.1002/jia2.26394)
Supplement: Supplementary file 3 — File S3: Early Adopter Codebook [file JIA2-27-e26394-s002.docx]

**Codebook: Early Adopters**

HIV Journey Experience

Considerations and Uptake

Experience of Long Acting

Psychosocial Impact

Alternative Formulations

Suggestions for Improvement

Quotable Quotes

Use memo function for observations related to:

Transportation

Stigma (HIV or other)
