# Supplementary material for: Learning from the first: a qualitative study of the psychosocial benefits and treatment burdens of long‐acting cabotegravir/rilpivirine among early adopters in three U.S. clinics
Source: J Int AIDS Soc. 2024 Nov 20;27(11):e26394. doi: 10.1002/jia2.26394 (PMC11578930; doi:10.1002/jia2.26394)
Supplement: Supplementary file 4 — File S4: Early Adopter Analysis Template [file JIA2-27-e26394-s003.docx]

**Informant:** **Date:**

**Primary Reviewer: Secondary Reviewer:**

**OBJECTIVES:** The aim is to consider holistically the experience of HIV and its management within the informant's lived experience, including the decision and impacts of transitioning to LAI-ART.

**INSTRUCTIONS:** Be specific and detailed in your reflections. Keep in mind that the goal is not to summarize the data but to *interpret* it.

**DEMOGRAPHICS:**
Age: XX | Gender: XX | Race/Ethnicity: XX | Sexual Orientation: XX | Education: XX | Housing: XX | Finances: XX

HIV Dx: XX | ART Start: XX | Adherence: XX | VS History: Ever/Never | Other Pills: XX | Other Injections: XX

Recent Drug Use: XX | Psych Dx: XX | Incarceration Hx: XX

VL @ Uptake: XX | Number of Injections: XX | Dosing Schedule: XX | Discontinuer: Yes/No

**STEP 1: CASE NARRATIVE:** In this section, the focus is on ***interpreting and narrating your story*** of this person’s experience. One way to frame this approach is to ask, “What is the point of this person’s story?”

**What were your reactions & takeaways from this case (3-6 sentences):**

*Briefly narrate your case. Who this person is holistically? What do their health and health care self-management strategies involve? How do they make decisions about LAI-ART? Do any tradeoffs arise from these decisions? What are the psychosocial impacts?*

**STEP 2: MAPPING IMPACTS:** In this section, the focus is on ***mapping and describing the unfolding of psychosocial impacts*** in this person’s experience.

**Describe The Process(es) Through Which The Psychosocial Impact(s) Unfolded (1-2 paragraphs max):**

*Consider essential conditions, mediating factors, contextual complexities, temporal aspects, and the interplay between other impact processes. Please consider the acuity or awareness of changes “before and after” LAI-ART and implications for persistence. Areas in which to consider changes include: HIV care and treatment, physical health, mental health, interpersonal relationships, clinic/provider relationships, and overall quality of life.*

**Narrative Describing “Links” and “Patterns”:**

**STEP 3: CASE SYNTHESIS**

In this section, the focus is to ***critically compare and contrast*** this person’s experience with what we have learned from other cases.

**How does this case align or diverge with emerging themes regarding psychosocial impacts? (1-2 paragraphs max)**

*Analyze the specific ways this person's feelings and relationships were influenced by transitioning to LAI-ART and compare them to what we have learned from other cases. Please attend to anything that still feels unclear with regard to psychosocial impact for this case.*
